# Effect of *Boswellia papyrifera* on cognitive impairment in multiple sclerosis

**Published:** 2014-07-04

**Authors:** Behnaz Sedighi, Abbas Pardakhty, Hoda Kamali, Kaveh Shafiee, Bahar Naz Hasani

**Affiliations:** 1 Department of Neurology, School of Medicine, Neurology Research Center, Kerman University of Medical Sciences, Kerman, Iran; 2 Department of Pharmacology ,School of Pharmacy, Pharmaceutics Research Center, Neuropharmacology Institute, Kerman University of Medical Sciences, Kerman, Iran

**Keywords:** Multiple Sclerosis, *Boswellia papyrifera*, Cognitive Impairment, Brief International Cognitive Assessment for Multiple Sclerosis, Traditional Medicine

## Abstract

**Background: **Cognitive impairment is one of the most crucial disorders among multiple sclerosis (MS) patients. Since MS is an inflammatory disease and *Boswellia papyrifera* has anti-inflammatory effects, the influence of *B. papyrifera* on cognitive impairment in MS patients has been investigated in the present study.

**Methods:** In this clinical trial, 80 MS patients who referred to the clinic of Shafa Hospital, Kerman, Iran were selected. Having completed a written consent form, patients with relapsing remitting MS, with no occurrence of a new attack throughout 1 month before the study, with no pregnancy or breastfeeding entered the study. The patients were randomly divided into two groups; then Brief International Cognitive Assessment for MS (BICAMS) test was carried out. One group received *B. papyrifera* (capsule 300 mg, twice a day) while the other group received placebo with the same dose for 2 months. After 2 months of treatment, BICAMS was redone and changes were analyzed. The significant change value on the before-after BICAMS points were considered to be 8, 13, and 7 points for the symbol digit modality test (SDMT), the California verbal learning test (CVLT), and the brief visual-spatial memory test revised (BVMT-R), respectively.

**Results: **The patients’ mean age was 36.58 8.50 years. The mean duration of disease was 7.41 4.13 years. About 84.2% (n = 64) of the patients was female. In the BVMT-R, 13 patients (34.2%), who had already taken *B. papyrifera*, were shown to have significant improvement compared to the placebo group with no improvement (P < 0. 001). About 12 and 8 patients in the treatment and placebo groups in the SDMT, respectively (P = 0.200) and 17 and 12 patients in the treatment and placebo groups in the CVLT, respectively (P = 0.170) had significant change values.

**Conclusion:** *B. papyrifera* showed significant improvement in visuospatial memory, but had no effect on verbal memory and information processing speed.

## Introduction

Multiple sclerosis (MS) is an inflammatory disease of the central nervous system, leading to demyelination and neurodegeneration in most patients.^1^^,^^2^ Cognitive impairment is regarded as one of the most important disorders in MS patients with prevalence of about 43-70%.^3^ Mild to moderate cognitive abnormalities are not usually apparent through a routine office visit and need more sophisticated neuropsychiatric assessment.

Cognitive impairment in MS affects patients’ quality of life. It is probably the most important determinant of employment status and associated societal costs, and also adversely affects driving safety, household task completion, social activity, physical independence, rehabilitation progress, coping, treatment adherence, and mental health.^4^

Cognitive impairment would negatively impact on all disease stages and in all MS subtypes. Full clinical cognitive assessment is expensive, requiring expert staff and special equipment. In addition, test versions and normative data are not available for all languages and cultures.^5^

Traditional medicine is getting more attention in MS management. *Boswellia* is a resinous extract from the trees of the genus *Boswellia*. A number of independent clinical studies support the anti-inflammatory and anti-arthritic properties of *Boswellia *extracts.^6^^-^^12^ Recent studies have shown that *Boswellia* has neuroprotective properties^13^^,^^14^ and can enhance the structural formation of new nerve networks, since every neurodegenerative disease like MS involves the destruction of these networks including memory loss and loss of cognitive ability; thus, *Boswellia* can be considered in that disease.^15^

*Boswellia papyrifera* (*BP*) is an Iranian traditional medicinal herb.^16^ Some initial studies in Iran have reported positive effects of *BP* on the enhancement of cognition in rats.^17^^,^^18^ Unfortunately, no clinical trial has been ever performed for the investigation of *BP* effects on the cognitive impairment in MS patients.

Various batteries of tests are designed to assess cognitive impairment in MS patients including brief repeatable battery of neuropsychological tests, Minimal Assessment of Cognitive Function in MS (MACFIMS) and MS Functional Composite.

An International Expert Consensus Committee recommended a brief battery of tests for cognitive evaluation in MS in 2012. The Brief International Cognitive Assessment for MS (BICAMS) battery includes tests of mental processing speed and memory.^4^

BICAMS is optimized and reliable for small centers, which may not have neuropsychological training and were constructed to maximize international use.^4^

Tests like the paced auditory serial addition test are primarily used in research trials. A 15-min battery of three tests assessing functions most commonly impaired in MS patients is now being validated and is called BICAMS.^19^

BICAMS test is optimized for small centers, with few staff members, which might not have neuropsychiatric training.^4^ It encompasses the symbol digit modality test (SDMT), first five recall learning trial of the California verbal learning test (CVLT-II) and the first three recall trials of the brief visual-spatial memory test revised (BVMTR).^5^

Therefore, it is endeavored to examine the effectiveness of *BP* on cognitive impairment of MS patients using BICAMS test.

## Materials and Methods

This study is a randomized, double-blinded, and placebo-controlled study (IRCT2013070813911N1) on MS patients who had referred to the MS clinic of Shafa Hospital, Kerman, Iran. This hospital is a referral center for MS patients.

***Patient selection***

*Inclusion criteria*

Relapsing remitting MS patients, MS patients under interferon therapy, patients who filled a written consent form to participate in the trial, patients with sufficient literacy for doing BICAMS test.

*Exclusion criteria*

Patients receiving corticosteroid over the 1 month before beginning of the study, patients with severe motor deficit and were unable to do the BICAMS, pregnant or breast feeding women, occurrence of new MS attack when the patient was under study.

After explaining all stages of the process of the study and all aspects of drugs (Including its advantages and its side-effects) to the patients, written consent has been taken. Then BICAMS battery was carried out on the patients. This battery was validated by MS Research Center of Sina Hospital of Tehran University of Medical Sciences, Tehran, Iran.^20^

After carrying out BICAMS battery, the patients were accidentally randomized into two different groups. One group received a capsule containing 300 mg of *BP*, twice a day while the placebo group received a capsule with the same size, color, shape, and dose, but without *Boswellia* active ingredient. Both capsules were made in the Pharmacology Research Center of Kerman University of Medical Sciences, Kerman, Iran. The patients were asked to continue their routine treatment, which was matched between the two groups and also to inform the research team in case of receiving another treatment during the follow-up period.

The patients would be withdrawn from the study if any side-effect or MS attack were occurred. After 2 months of the treatment period, BICAMS was redone and data were analyzed.

We considered the significant changes in the BICAMS batteries providing that there were a change of 8, 13, and 7 points in SDMT, CVLT, and BVMT-R, respectively.^5^

The present study had been approved by Ethics Committee of Kerman University of Medical Sciences (Ethical Code: K/91/379).

## Results

In the present study, 80 MS patients have been randomized into two groups after BICAMS battery was carried out. Two patients, in both treatment and placebo groups, were excluded from the study due to diarrhea and a MS attack. Therefore, 38 patients in each group were able to finish the study. After taking the drug for 2 months and reassessment of BICAMS battery, following results were observed.

The patients’ mean age was 36.58 8.50 years however the mean age in the treatment and placebo groups was 36.21 8.25 and 36.95 8.90 years, respectively. The overall mean duration of MS was 7.41 4.13 years with 6.87 3.80 years and 7.95 4.36 years for the treated and placebo groups, respectively. About 84.20% (n = 64) of the patients in this study were female. There was no significant difference between the two groups in terms of age (P = 0.71), sex (P = 0.34), and the disease duration (P = 0.25).

BICAMS battery changes were considered as significant if SDMT changed 8 points, CVLT, 13 points, and BVMT-R, 7 points.

We observed that 12 patients (31.6%) from the treatment group and 8 patients (21.1%) from the placebo group had a significant change value in SDMT test.

CVLT test also showed a significant change in 17 and 12 patients (44.7% and 31.6%) in the treatment and placebo groups, respectively.

BVMT-R was improved significantly in 13 patients (34.2%) who took BP compared to the placebo group which had no improvement at all.

Although the number of patients who improved after receiving the drug in the treatment group was more than the placebo group in CVLT and SDMT, such a difference was not statistically significant (P = 0.2000 for SDMT, P = 0.1700 for CVLT); regarding BVMT-R, our findings were statistically significant (P < 0.001).

Notably, it seemed that sex might be influential. All patients whose SDMT improved were female in contrary to males. The male patients did not improve in that test (P = 0.024). 

**Figure 1:**
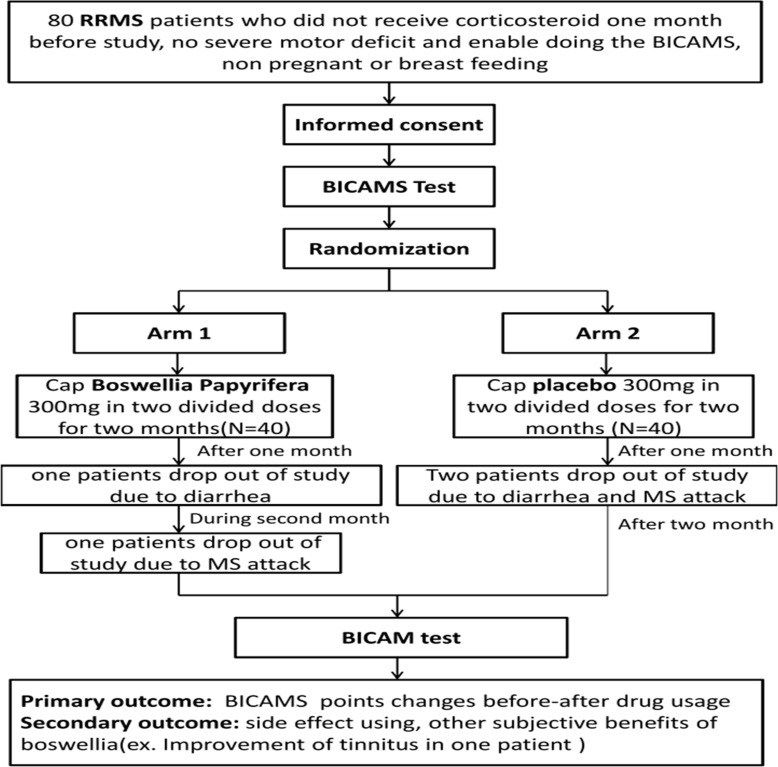
The flow chart of study

**Figure 2:**
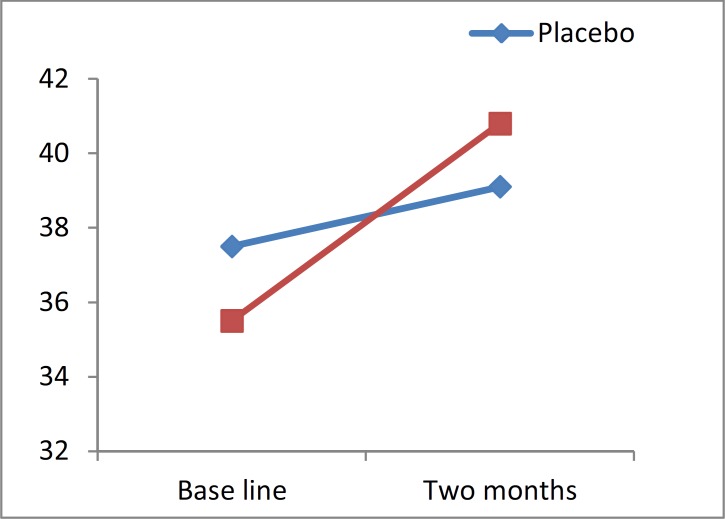
Symbol digit modality test changes of value versus time

**Figure 3:**
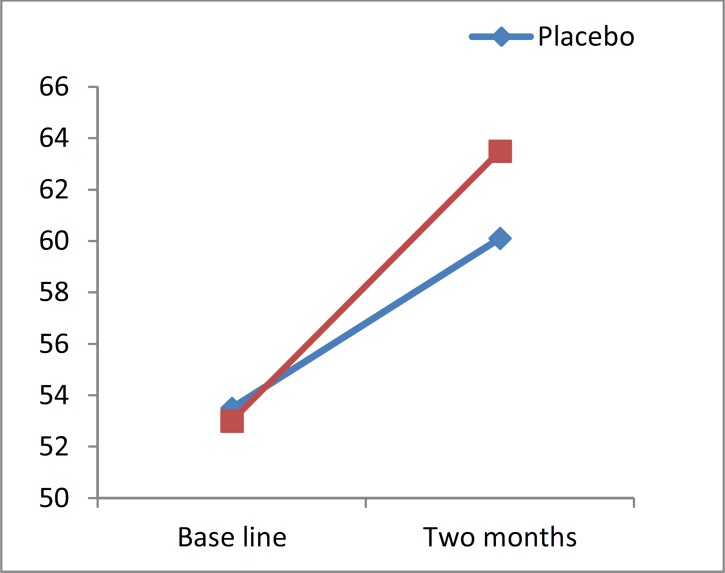
California verbal learning test changes of value versus time

**Figure 4:**
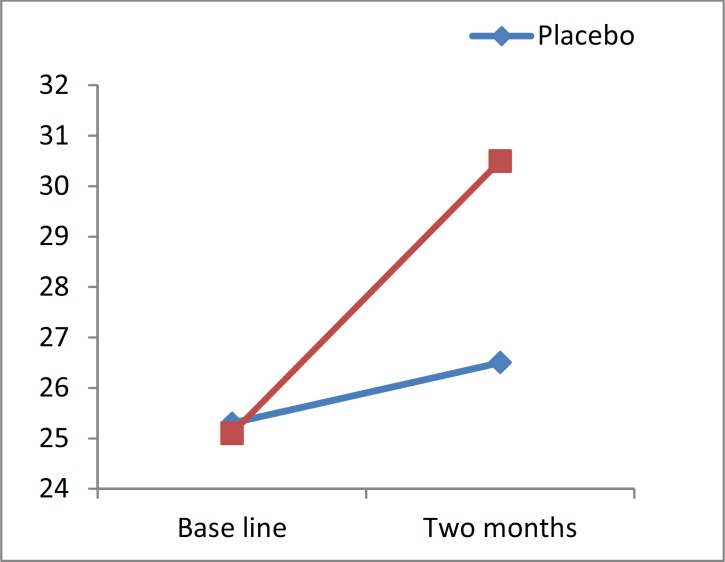
Brief visual-spatial memory test revised changes of value versus time

## Discussion

MS is an inflammatory and neurodegenerative disease that causes cognitive and memory impairment. Oxidative stress accompanied by depletion of endogenous antioxidant level is known as an etiology of cognitive impairment.

Since *Boswellia* has an antioxidant effect, it has been suggested to be useful in the improvement of the cognitive impairment.^13^^,^^14^ We were not able to find any study in which the effect of *Boswellia* on cognitive impairment of MS patients had been investigated by using BICAMS battery. In this study, the patients who used *Boswellia* showed a significant improvement in the visual-spatial memory as measured by BVMTR.

Our findings would relatively confirm the previous results by Farshchi et al.^17^ and Mahmoudi et al.^18^ In that study, it was shown that BP had a significant effect on improvement of the spatial learning in rats.

Another study conducted on healthy volunteers,^21^ showed that although the verbal and logical memory improved by using BP, the visual memory did not change. These findings are in contrast with the present results. Our study also showed that although BP is probably effective on the improvement of visual-spatial memory (BVMT-R), it is not effective on the auditory-verbal learning ability (CVLT) in MS patients.

Another reason that BP did not improve analyzed cognitive skills in the auditory-verbal learning test in our study might be due to its dose-dependent effects. This dose-dependent effect was reported by Farshchi et al.^17^ and Mahmoudi et al.^18^ They observed that the effect of BP on rat’s visual memory was dose-dependent. Hence, we believe that BP’s effects on the cognitive tests of this study should be carried out with various doses.

The longer BP is used, the more effective it may be on cognitive improvement. Therefore, it is possible that by increasing treatment duration, the effects of this drug on other cognitive skills may be more prominent.

We recommend that further studies be done to evaluate the effect of BP on the improvement of cognitive impairment in MS patients.

## Conclusion

To conclude, *Boswellia* might significantly improve visual-spatial memory. We recommend further investigations on various dosage and longer treatment duration of BP.

## Notes

**How to cite this article: **Sedighi B, Pardakhty A, Kamali H, Shafiee K, Hasani BN. Effect of *Boswellia papyrifera* on cognitive impairment in multiple sclerosis. Iran J Neurol 2014; 13(3): 149-53.
